# A cluster randomised controlled trial evaluating the effectiveness of eHealth-supported patient recruitment in primary care research: the TRANSFoRm study protocol

**DOI:** 10.1186/s13012-015-0207-3

**Published:** 2015-02-03

**Authors:** Nikolaos Mastellos, Anna Andreasson, Kit Huckvale, Mark Larsen, Vasa Curcin, Josip Car, Lars Agreus, Brendan Delaney

**Affiliations:** Department of Primary Care and Public Health, School of Public Health, Imperial College London, 3rd Floor Reynolds Building, St Dunstan’s Road, London, W6 8RP UK; Centre for Family Medicine, Karolinska Institutet, Alfred Nobels Allé 12, Huddinge, 14183 Sweden; Department of Primary Care and Public Health Sciences, School of Medicine, King’s College London, 7th Floor Capital House, 42 Weston Street, London, SE1 3QD UK

## Abstract

**Background:**

Opportunistic recruitment is a highly laborious and time-consuming process that is currently performed manually, increasing the workload of already busy practitioners and resulting in many studies failing to achieve their recruitment targets. The Translational Medicine and Patient Safety in Europe (TRANSFoRm) platform enables automated recruitment, data collection and follow-up of patients, potentially improving the efficiency, time and costs of clinical research. This study aims to assess the effectiveness of TRANSFoRm in improving patient recruitment and follow-up in primary care trials.

**Methods/design:**

This multi-centre, parallel-arm cluster randomised controlled trial will compare TRANSFoRm-supported with standard opportunistic recruitment. Participants will be general practitioners and patients with gastro-oesophageal reflux disease from 40 primary care centres in five European countries. Randomisation will take place at the care centre level. The intervention arm will use the TRANSFoRm tools for recruitment, baseline data collection and follow-up. The control arm will use web-based case report forms and paper self-completed questionnaires. The primary outcome will be the proportion of eligible patients successfully recruited at the end of the 16-week recruitment period. Secondary outcomes will include the proportion of recruited patients with complete baseline and follow-up data and the proportion of participants withdrawn or lost to follow-up. The study will also include an economic evaluation and measures of technology acceptance and user experience.

**Discussion:**

The study should shed light on the use of eHealth to improve the effectiveness of recruitment and follow-up in primary care research and provide an evidence base for future eHealth-supported recruitment initiatives. Reporting of results is expected in October 2015.

**Trial registration:**

EudraCT: 2014-001314-25

## Introduction

### Background

Recruitment to primary care trials is a particularly challenging process with most studies struggling to reach their recruitment targets. In fact, a survey of authors of 39 published primary care trials found that only 29% of UK primary care studies achieved their recruitment targets within the agreed timeframe, with 70% requiring additional time to recruit the predefined number of participants [1]. This is very worrying considering that clinical trials succeed or fail based on whether they manage to recruit the sufficient number of participants to enable researchers to generate accurate results to precisely and reliably answer the question at hand. Recruitment failings can result in studies lacking statistical power to produce significant results, increasing the risk that an effective health intervention will be abandoned before its real impact has been demonstrated [2]. Consequently, improving the recruitment process in clinical trials is extremely important for the future of medical research.

Evidence suggests that the most effective method of recruitment in primary care is opportunistic recruitment [3]. This involves approaching eligible primary care patients when they visit their general practitioner (GP) and inviting them to take part. Opportunistic recruitment takes place in a highly stressful and time-pressured clinical environment and relies on busy GPs directly inviting patients within the consultation [4]. As a result, this method can be very challenging and slow with practitioners often failing to translate the initial enthusiasm into recruitment targets [5].

The increased adoption of electronic health records (EHR) systems in primary care provides the opportunity for real-time identification of eligible participants through the use of clinical trial alert systems [6-9]. Such systems notify GPs about eligible patients during the consultation allowing them to immediately discuss the trial with the patient to enable instant recruitment, thus negating the need for laborious effort on the part of the practitioner, patient or researcher [10]. The Translational Medicine and Patient Safety in Europe (TRANSFoRm) project team has developed a suite of software tools and underlying infrastructure to support patient recruitment to primary care studies across multiple sites and countries, potentially saving time for busy practitioners and patients in the consultation room [11]. This protocol describes a cluster randomised controlled trial evaluating the effectiveness of the TRANSFoRm system in improving patient recruitment and follow-up in primary care trials.

**Figure 1:**
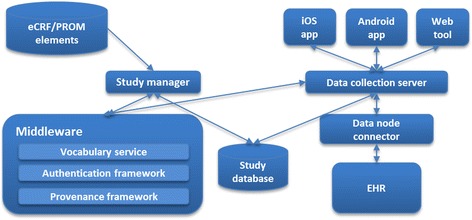
**TRANSFoRm system components.**

The TRANSFoRm system supports secure, provenance-enabled design, deployment and collection of patient-reported outcome measures (PROM) and electronic case report forms (eCRF) through web and mobile applications as well as EHR systems in the primary care centres where the trials are taking place, as shown in Figure 1. The GP-facing tools enable automated identification, eligibility check, randomisation, baseline and follow-up data collection and reporting of data in clinical studies, while the patient-facing tools support collection of patient-reported data through mobile or web applications.

**Figure 2:**
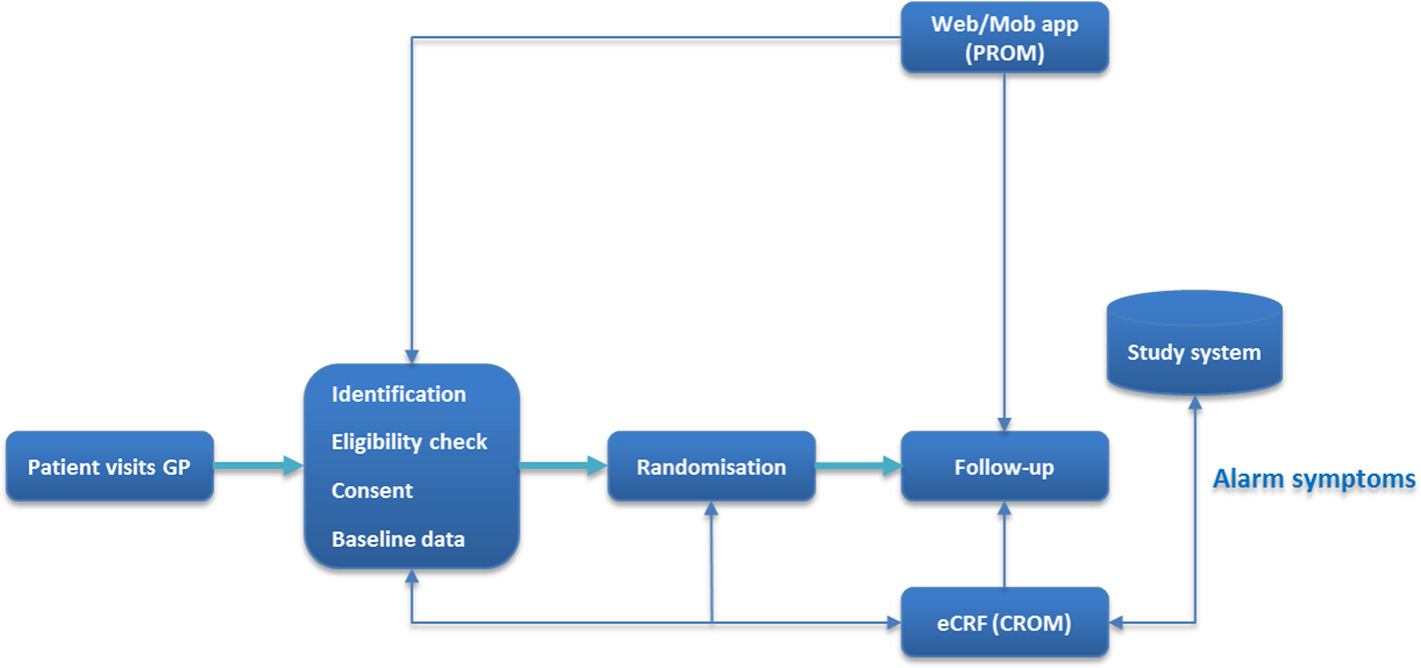
**TRANSFoRm clinical trial workflow.**

Figure 2 shows the TRANSFoRm-supported clinical trial workflow, starting with a patient visiting their GP for their consultation. Potential participants are identified using a clinical trial alert tool that notifies the GP that the patient fulfils specific inclusion criteria, after checking for diagnoses, symptoms and/or treatment prescriptions. Once identified, the GP can check for the first set of inclusion and exclusion criteria and, if the patient is still suitable, collect informed consent for further screening. Next, clinical data from the patient’s electronic record is extracted to populate fields in the eCRF. The system alerts the practitioner about any missing data and presents the study dataset for final approval. PROM data is incorporated into the eCRF via a web or mobile application used by participants to record symptoms, signs and other self-reported outcomes. The system randomises enrolled patients and reports allocation instantly so that the study intervention can be initiated without delay. Participants are then followed up using the mobile or web applications and the eCRF tool at GP visits. Following GP approval, the study dataset is delivered to the researcher through a secure data transport mechanism that communicates with TRANSFoRm connector tools on the participating EHR.

The development of the clinical study workflow has been structured around a clinical study which acts as a use case for the evaluation of TRANSFoRm. The gastro-oesophageal reflux disease (GORD) study employs a randomised controlled trial design to answer the following question: “*What gives most symptom relief and improvement in quality of life in patients with GORD, on demand or continuous use of proton pump inhibitors?*”. Participants will be patients with GORD, aged 18–65 years old. The study will take place in five countries and will involve 16-week recruitment and 8-week follow-up. The follow-up time was chosen because proton pump inhibitor (PPI) treatment is known to have an effect after 2 weeks. Therefore, 8 weeks should be sufficient to detect any changes in symptoms and quality of life. The role of TRANSFoRm in this study is to identify prevalent and incident cases of GORD, randomise patients to on-demand or continuous use of PPI and follow up these patients using mobile or web applications and eCRF completed by GPs at primary care visits. Further details about the clinical study will be provided in a separate publication.

### Aim and objectives

The study described in this protocol aims to assess the effectiveness of TRANSFoRm in patient recruitment compared to standard GP-led opportunistic recruitment. The study hypothesis is that TRANSFoRm will increase patient recruitment by 15%, from 20% to 35%, compared to standard practice.

The objectives of the study are:

1. To evaluate the effectiveness of TRANSFoRm in recruiting patients to clinical trials.
2. To assess its effectiveness in collecting complete baseline and follow-up data.
3. To compare the costs of recruitment in the TRANSFoRm and control arms.
4. To explore practitioner and patient experience and acceptability of the system.

## Methods/design

### Trial design

**Figure 3:**
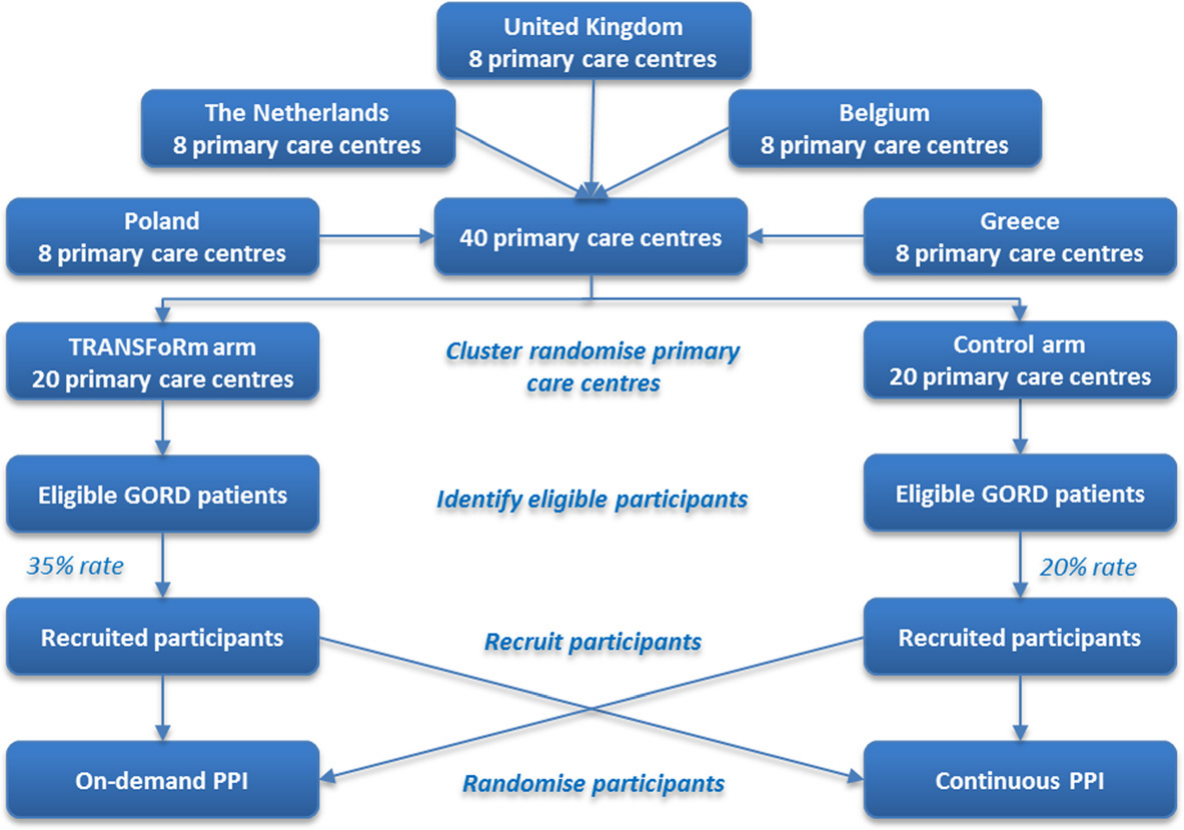
**Study design—a two-arm cluster randomised controlled trial comparing TRANSFoRm with standard GP-led opportunistic recruitment.**

We plan to conduct a 24-week, multi-centre, parallel-arm cluster randomised controlled trial of TRANSFoRm recruitment compared to GP-led opportunistic recruitment. The intervention arm will use the TRANSFoRm tools for participant recruitment, baseline data collection and follow-up. The control arm will rely on standard GP-led recruitment using web-based CRFs for baseline data collection and paper-based questionnaires for follow-up. The study will run in five countries (United Kingdom, The Netherlands, Belgium, Poland, and Greece) with eight primary care centres in each country, giving a total of 40 centres. Of these, 20 will be randomised to the TRANSFoRm arm and 20 to control on a 1:1 allocation ratio. Potentially eligible participants will be identified and recruited using TRANSFoRm or standard GP recruitment, with the hypothesis being that TRANSFoRm will yield 35% recruitment rate compared to 20% for the standard method. Participants will be finally randomised to on-demand or continuous use of PPI as part of the clinical protocol for the GORD study (Figure 3).

### Participants

The intervention will be implemented at the primary care centre level. To be eligible for participation, primary care centres should use an EHR system compatible with TRANSFoRm, provide written agreement to participate in the GORD study, agree to participate in whichever arm they are randomly allocated, have a list size of over 2,000 patients and be able to identify at least 100 eligible participants over the 16-week recruitment period. Primary care centres lacking an appropriate EHR system or centres that have migrated from paper-based record keeping within the last 12 months will be excluded. Primary care centre characteristics, such as list size and GORD prevalence, will be gathered from GP database audit to inform recruitment. Local project partners will liaise with the study coordinator to recruit care centres that meet the above criteria.

Study participants will be GPs located in primary care centres receiving the TRANSFoRm intervention or standard recruitment method, and GORD patients using the web/mobile- or paper-based method for recording self-reported data. To be eligible for participation, patients should be GORD cases with heartburn and/or acid regurgitation that need PPI treatment, aged 60–85. Pregnant women, patients with a myocardial infraction within 6 months of the study commencement or patients with serious comorbidity will be excluded from the study.

Informed consent will be sought from all sites. Primary care centres will provide informed consent as a unit. This may vary from all GPs signing the consent form to one GP being authorised to sign on behalf of the centre. Patients will consent in accordance with the requirements of the GORD trial. The participating centres will receive €17 compensation for each correctly enrolled patient. Patients will not receive any incentive for taking part. Participants are not expected to experience any harm as a result of their participation in the evaluation study, as their involvement is restricted to completing the PROM questionnaires. Despite including health information, it is unlikely that these questionnaires will address any sensitive issues.

### Setting

We aim to recruit 40 urban, suburban and rural primary care centres in and around five European sites: London (United Kingdom), Utrecht (The Netherlands), Antwerp (Belgium), Wroclaw (Poland) and Heraklion-Crete (Greece). A dedicated member of the study team will liaise with local coordinators in each country to recruit eligible practices. Detailed practice characteristics will be provided in future publications.

### Intervention and control

Primary care centres allocated to the intervention arm will use the TRANSFoRm tools for participant recruitment, baseline data collection and follow-up. When visiting their GP, potentially eligible patients will be flagged by the system so that their GP can check for the first set of inclusion and exclusion criteria and, if appropriate, collect informed consent for further screening. Next, clinician-reported outcome measures (CROM) data from the patient’s health record will populate fields into the patient’s eCRF. The system will notify the GP about any missing data. Upon GP approval, the system will randomise patients to on-demand or continuous use of PPI. Participants will then receive information on how to fill out the PROM fields on their smartphone or the web. When participants return for the follow-up visit after 8 weeks, as stipulated in the clinical protocol, the CROM data is activated; the GP checks all prefilled fields and adds any missing data. If the participant does not attend the second visit, the GP will receive a reminder. PROM data is provided by the participants within 3 days from the second visit using the mobile/web application. Participants will receive reminders 2, 3 and 4 days after the visit, if the self-reported data is not provided.

Control centres will use standard GP-led opportunistic recruitment and data collection methods. Patients will be identified as potentially eligible by their GP after checking their health record during the consultation. The GP will check for the exclusion criteria, and, if the patient is still eligible, they will provide them with information about the study. Upon patient consent, the GP will complete the CROM fields and the patient will be randomised to on-demand or continuous use of PPI following the randomisation design for the GORD study (i.e. block randomisation containing four blocks based on age and gender). The patient will receive a questionnaire to complete either at the primary care centre or at home. Patients will be followed up using paper-based questionnaires.

### Primary outcome

The primary outcome measure is the proportion of eligible patients successfully recruited using the TRANSFoRm method compared to standard GP-led opportunistic recruitment. A record of all identified, screened and successfully recruited patients will be kept using data from GP systems. The percentage of those recruited in the trial will be compared to identify whether TRANSFoRm yields a higher number of recruited patients compared to standard GP recruitment.

### Secondary outcomes

Secondary outcomes will include the proportion of GORD cases recruited with complete baseline and follow-up data, as well as the proportion of those giving informed consent to participate but later withdrawing from the study. All electronic or paper-based CRFs completed by the recruited patients in each group will be reviewed, and retention data will be collected to examine whether there is a difference in the percentage of patients with complete data and the numbers of participants withdrawn or lost to follow up among the two arms.

### Economic evaluation

A cost analysis will estimate the costs of recruitment using the TRANSFoRm system and the standard method. A similar approach was adopted in a previous study comparing recruitment methods across sites [12]. The analysis will focus on the direct costs incurred to the primary care centre during the implementation and operation phases. Patient and third party costs will not be included. Trial administration costs, including labour and material costs and personnel time spent will be identified and combined with recruitment data to model the average cost per site and enrolled participant. Non-recurring costs will spread out over 15 years and will be discounted at a 3.5% rate. If TRANSFoRm results in increased recruitment but is also more costly, the cost per additional participant will be estimated to assess whether this excess cost is acceptable.

### Technology acceptance survey

User acceptance of the TRANSFoRm tools will be measured in a centrally coordinated technology acceptance survey. The Technology Acceptance Model (TAM) questionnaire is a validated instrument for collecting *post hoc* perceptions of a technology artefact segregated into two domains: usefulness and ease of use [13-15]. Data will be collected retrospectively through an electronically distributed questionnaire to all intervention arm participants.

### User experience interviews

Clinician and patient experience with the TRANSFoRm tools will be assessed in follow-up, semi-structured, in-depth interviews with a purposive sample of interesting cases (i.e. GPs and patients with positive and negative experiences who demonstrated high/low usage patterns during the study). This interview study aims to assist interpretation of quantitative data generated by the main evaluation study by exploring: i) methods of recruitment and follow-up (e.g. web/mobile- vs. paper-based PROM); ii) reasons for any observed variations in recruitment performance across sites; iii) barriers and facilitators to successful recruitment; and iv) areas for improvements.

### Sample size

We estimated the average number of eligible GORD patients per primary care centre to be at least 100 during the 16-week recruitment phase, based on a conservative GORD prevalence of 10% [16] and a list size of 2,000 patients. We conducted a binominal power calculation taking into account the variation in centre characteristics. This showed that 536 participants across 40 centres, 20 per arm, will give the study 90% power to detect a 15% increase in recruitment, from 20% to 35%, using a conservative 5% intra-cluster correlation coefficient, at 5% significance level. Modelling conducted for the clinical study suggests that this will offer excess power to detect a clinically relevant change in self-rated health scores (±0.25 Reflux Disease Questionnaire points) between treatment groups.

### Randomisation, allocation and blinding

Primary care centres will be assigned to the TRANSFoRm or control arm on a 1:1 allocation ratio. The allocation sequence will be computer-generated using a within-country permuted random block design with blocks of two matched for list size, GORD prevalence, location, ratio of GORD appointments per week to list size and ratio of GPs to list size. The study is unblinded as it is impossible to blind primary care centres or investigators to the intervention. However, the allocation will not be shared with those involved in the data analysis to maintain objectivity and minimise bias.

### Statistical methods

The percentage of patients recruited to the study for the intervention and control groups will be calculated using descriptive statistics with 95% confidence intervals. These will be presented separately for each site, country and overall. The proportion of eligible participants recruited will be compared between groups using logistic regression, adjusted for clustering by centre by using generalised estimating equations (CI 95%, *P* < .05). Between-group differences in recruitment rate will be analysed using chi-square tests. This method will be also applied to compare the percentage of participants with complete data and the percentage of eligible patients who were recruited but later withdrew or lost to follow up.

Descriptive statistics will be used to outline the relative proportion of participants who were satisfied or dissatisfied with the TRANSFoRm tools as per item on the TAM questionnaire. Quantitative data will be analysed in STATA (v13) using an intention-to-treat approach.

Data from the user experience interviews will be audio-recorded, transcribed verbatim and analysed thematically using Ritchie and Spencer’s framework analysis approach [17].

The study has been designed and will be reported in CONSORT-compliant format [18].

### Handling and storage of data

The study will be conducted according to good clinical practice standards. The handling of personal data will comply with the Personal Data Protection Act 2012 and local data protection regulations. Data collected from EHR systems and mobile/web applications using the TRANSFoRm system will be stored in a secure site hosted by Custodix (a private company specialising in data protection solutions in eHealth). TRANSFoRm has implemented technical measures to ensure that identifiable personal data is protected and that study data in the TRANSFoRm study system is protected by access authorisation and pseudonyms. Data for the control group will be transcribed from the paper-based forms via a web-based clinical data capture system hosted on a secure data repository at King’s College London. At the end of the study, all data will be securely transferred to Custodix to create the analysis dataset. This will only contain pseudonymised data. After analysis and reporting, data will be archived at the Karolinska Institutet in Stockholm for 15 years.

## Discussion

Recruitment failures in primary care are common due to the complexity of recruiting patients in this setting. To date, the most effective method for recruiting patients to primary care trials is opportunistic recruitment. This is performed manually using paper- or web-based case report forms and relies on busy GPs actively approaching potentially eligible patients in the consultation room and inviting them to take part. In an increasingly demanding healthcare system, such methods are highly time-consuming, and as a result, many studies fail to achieve their recruitment targets or to even recruit a single patient [1,19,20]. Recent advancements in information technology have enabled researchers, technologists and clinicians to collaborate with the aim to develop systems that can overcome these challenges and support effective recruitment. The TRANSFoRm system aims to transform patient recruitment and follow-up in primary care by automating the processes for identifying, recruiting and following up patients, potentially saving time for health professionals, which can be used instead in care delivery. The study described in this protocol will evaluate the effectiveness of TRANSFoRm in improving the efficiency, time and costs of recruitment and data collection. The study will provide an evidence base for eHealth-supported recruitment and follow-up in primary care. Participant recruitment is scheduled to commence in February 2015 and be completed by May 2015. The scheduled end of follow-up data collection is July 2015. Reporting of results is expected in October 2015.

The study design has been tailored to the objectives of the evaluation and the characteristics of the GORD study. The evaluation focuses on the socioeconomic aspects of the system. The main goal is to ensure that TRANSFoRm can improve the efficiency, time and costs of conducting clinical research in primary care. The evaluation strategy draws on valid and reliable methods which have been widely used in health informatics research to assess the effectiveness of eHealth interventions [21]. The study will use quantitative and qualitative data collection techniques that enable triangulation of findings adding to the internal validity of the study. It will recruit urban, suburban and rural primary care centres in five countries and will employ culturally diverse study participants increasing the sample representativeness and thus external validity.

The evaluation will include patients with GORD, and otherwise eligible for the study, for whom a single clinic appointment was made during the recruitment period. This measure guards against the possibility that participating centres embark on an active programme of recruitment of potential participants rather than, as instructed, relying on opportunistic presentation. Local EHR and appointment datasets will be used to validate this process. An in-depth audit will be undertaken of any centre where the appointment rate lies more than two standard deviations from the within-country mean. In all other centres, a random selection of appointments created for GORD patients during the recruitment period will be audited by an evaluation team member in liaison with a local collaborator.

The study has some weaknesses. The scope of the economic analysis is limited to the care centre level. This practically means that we cannot assess the costs and benefits for all stakeholders in the project. However, patients will be able to provide qualitative feedback on the impact of the TRANSFoRm tools in follow-up interviews and a TAM survey. Another limitation is that the study timeline is relatively short to evaluate the long-term effects of the intervention, despite being sufficient to recruit the required number of participants and collect data to explore the evaluation aims. Considering that technology adoption is a complex process [22,23], the time from implementation to evaluation is relatively short to assess the performance of the TRANSFoRm system.

The protocol provides a reproducible process for conducting a summative evaluation of the TRANSFoRm system based on the GORD clinical trial. The evaluation study focuses on assessing the effectiveness of TRANSFoRm-supported recruitment and follow-up in primary care research compared with standard GP-led opportunistic recruitment. The study should provide useful information about the use of information technology to improve the efficiency, time and costs of recruitment and follow-up in primary care and is expected to provide an evidence base for future eHealth-supported recruitment initiatives.

## Abbreviations

CROM: Clinician-reported outcome measures
eCRF: Electronic case report forms
EHR: Electronic health records
GORD: Gastro-oesophageal reflux disease
GP: General practitioner
PPI: Proton pump inhibitor
PROM: Patient-reported outcome measures
TAM: Technology Acceptance Model
TRANSFoRm: Translational Medicine and Patient Safety in Europe
